# Causes of multidrug-resistant tuberculosis from the perspectives of health providers: challenges and strategies for adherence to treatment during the COVID-19 pandemic in Brazil

**DOI:** 10.1186/s12913-021-07057-0

**Published:** 2021-10-01

**Authors:** Ludmilla Leidianne Limirio Souza, Felipe Lima dos Santos, Juliane de Almeida Crispim, Regina Célia Fiorati, Sónia Dias, Alexandre Tadashi Inomata Bruce, Yan Mathias Alves, Antônio Carlos Vieira Ramos, Thaís Zamboni Berra, Fernanda Bruzadelli Paulino da Costa, Luana Seles Alves, Aline Aparecida Monroe, Inês Fronteira, Ricardo Alexandre Arcêncio

**Affiliations:** 1 Master of Science, Public Health Nursing Graduate Program, University of São Paulo at Ribeirão Preto College of Nursing, Avenida dos Bandeirantes, 3900, Monte Alegre, Ribeirão Preto, São Paulo, Brazil; 2 Postdoctoral Fellowship in the Interinstitutional Doctoral Program in Nursing, University of São Paulo at Ribeirão Preto College of Nursing, Ribeirão Preto, São Paulo, Brazil; 3 University of São Paulo at Ribeirão Preto Medical School at Ribeirão Preto, São Paulo, Brazil; 4 Universidade NOVA de Lisboa at National School of Public Health, Lisbon, Portugal; 5 Public Health Nursing Graduate Program, University of São Paulo at Ribeirão Preto College of Nursing, Ribeirão Preto, São Paulo, Brazil; 6 University of São Paulo at Ribeirão Preto College of Nursing, Ribeirão Preto, São Paulo, Brazil; 7 Global Health and Tropical Medicine, Instituto de Higiene e Medicina Tropical, Universidade NOVA de Lisboa, Lisbon, Portugal

**Keywords:** Tuberculosis, Multidrug-resistant tuberculosis, COVID-19, Qualitative research, Social determinants of health

## Abstract

**Background:**

Multidrug-resistant tuberculosis (MDR-TB) is a serious phenomenon on a global scale that can worsen with the COVID-19 pandemic. The study aimed to understand the perceptions of health professionals about MDR-TB, their strategies to ensure adherence to treatment and their challenges in the context of the COVID-19 pandemic in a priority municipality for disease control.

**Methods:**

We conducted a qualitative study and recruited 14 health providers (four doctors, three nurses, three nursing technicians, three nursing assistants and a social worker) working in a city in the state of São Paulo, Brazil. Remote semi-structured interviews were conducted with the participants. For data analysis, the thematic content analysis technique was applied according to the study’s theoretical framework.

**Results:**

The study revealed the causes of MDR-TB are associated with poverty, vulnerability, and social risk. A pre-judgement from the providers was observed, namely, all patients do not adhere due their resistance and association with drug abuse or alcoholism. The study also observed difficulty among health providers in helping patients reconstruct and reframe their life projects under a care perspective, which would strengthen adherence. Other issues that weakened adherence were the cuts in social protection and the benefits really necessary to the patients and a challenge for the providers manage that. The participants revealed that their actions were impacted by the pandemic and insecurity and fear manifested by patients after acquiring COVID-19. For alleviating this, medical appointments by telephone, delivery of medicine in the homes of patients and visits by health professionals once per week were provided.

**Conclusion:**

The study advances knowledge by highlighting the challenges faced by the health system with the adherence of patients with MDR-TB in a context aggravated by the pandemic. An improvement in DOT is really necessary to help the patients reframe their lives without prejudices, face their fears and insecurity, recover their self-esteem and motivate in concluding their treatment.

## Background

Tuberculosis (TB), an infectious disease caused by the bacillus *Mycobacterium tuberculosis*, remains a serious public health problem. Despite the advances made in controlling the disease in recent years, on a global scale, its prevalence has still reached critical levels [1]. According to estimates by the World Health Organization (WHO), TB is one of the 10 leading causes of death in the world and the main cause of death by a single infectious agent. In the last 5 years, it has occupied first place on the list of most deadly infectious diseases [2].

The TB situation becomes more serious as the advance of multidrug-resistant TB (MDR-TB) is observed, that is, resistance to at least two of the drugs used for treatment, isoniazid and rifampicin [3]. According to the WHO, it is estimated that a total of 500,000 cases developed resistance to rifampicin in 2020, and of these, 78% of patients developed MDR-TB [2].

MDR-TB has been a serious threat to health on a global scale [4], becoming even more complex when considered in the context of the COVID-19 pandemic. There is evidence that the meeting of these two diseases or even the simultaneous occurrence of these phenomena will be a “catastrophe” [5].

In Brazil, the main clinical form is acquired (95% of cases). This is due to the fact that patients are sensitive to the initial treatment drugs, which become resistant due to therapeutic discontinuities [6]. The WHO data show that these patients actually have 20 times more costs when compared to those with sensitive TB [2, 7].

Some determinants are related to MDR-TB, including individual characteristics (age, sex, and educational level), behavioral (substance abuse, smoking, and alcoholism), clinical (HIV), history of previous treatment, type of resistance, access to health services, reduction of social protection policies and the social contexts of poverty and privation [8]. All these socioeconomic and cultural determinants are related to adherence, with limited access to health services, and the patients do not have enough financial resources to support their treatment, which reflects in treatment defaults. Adherence is understood as a means to an end, an approach to maintaining or improving health, reducing the signs and symptoms of a disease [9].

In Brazil, in 2020, adherence, expressed by successful treatment, was much lower that the goals recommend by the WHO, accounting for 71% in reality when the expected was more than 85% among the sensible cases [2]. The cases where MDR-TB was observed were dramatic, with successful treatment ranging from 40 to 60% [10]. It can be become more complex, since there is evidence there was a decrease of 16% in the notification of new TB cases in 2020 as compared to 2019. However, it is not known yet how the pandemic may have influenced the adherence of patients, mainly because of its interference in the health workforce and health systems [11].

The determinants of MDR-TB should be understood, in addition to its biomedical aspects, incorporating the subjectivity of access, bonding, reception, health needs, and adherence, among others [12]. In addition to this understanding, the senses and experiences of health professionals who are at the forefront of care can contribute to advancement and equity in the context of care for MDR-TB [13].

Studies have been conducted to understand the challenges of TB care from the perspective of the professional, whether in terms of the quality of care [14] and/or professional and patient satisfaction [15]. However, we did not observe studies that (besides evidencing the causes of MDR-TB considering their universal symbolism among providers) also aggregated strategies for supporting the patients in following their treatment, mainly in a context absolutely affected by the COVID-19 pandemic due to interruptions of directly observed treatment (DOT), cuts in social benefits, and fear and insecurity among patients, who were already devasted by MDR-TB. Here, we have to apply the concept of a syndemic (not a pandemic), as some countries and/or studies on the subject have been mistakenly classified. A syndemic is understood as a set of interconnected health problems involving two or more complications that interact synergistically and contribute to the excessive burden of disease in a population [16].

Therefore, the aim of this study was to understand the causes of MDR-TB through perspectives from health professionals and the strategies adopted to improve adherence to treatment in a context affected by COVID-19 in the state of São Paulo, Brazil.

## Methods

### Study design

It was a qualitative study, which used the thematic content analysis technique [17] and was guided by the recommendations of the Consolidated Criteria for Reporting Qualitative Research (COREQ) [18].

### Theoretical framework

For the study, the theoretical framework for social determinants of health (SDH) was defined [19] in order to compare the results of the investigation and to allow incursions on the meaning of MDR-TB for health professionals. This framework leads to reflection on the social structure and therefore structural determinants of the concept of “social position” and of different and unequal opportunities, resulting from class and power divisions, which greatly influences health inequities. The social mechanisms to face social determinants result from political and human rights processes involving communities and the state itself [20].

The social conditions in which people live and work [20] stem from these forces, and the health-disease balance will be determined by the economic, cultural, environmental and biological dimensions [21] that occur within the framework of MDR-TB.

In micropolitics, a materialization of the intermediary social determinants is access to the structure of health services and adherence to care technologies, and in this sense, we take the work of Bertolozzi and colleagues [22] who conceptualized it not only as an unreduced act of personal volition but also a process associated with life, which depends on intermediaries (especially health professionals) who, in the person’s daily life and the organization of their health work processes, contribute to a patient’s access to health services from a perspective of care and not of disease control. Based on this concept, the principle of equity, which refers to the offer and organization of services according to a gradient of needs, and the willingness of professionals to operate practices in the territories in this logic, of social justice [23]. The issue of equity becomes more challenging, as COVID-19 imposes new work strategies and organizations of care on professionals, which lack further investigative depth [24].

### Study setting

The study was conducted in Ribeirão Preto, which is located in the interior of the state of São Paulo, Brazil. The choice of the scenario is justified, because it is a reference municipality in the treatment and monitoring of MDR-TB and classified as high risk [10]. For the treatment of MDR-TB cases, we have the Clinics Hospital of the Ribeirão Preto Medical School at the University of São Paulo (HCFMRP-USP) in the city and the additional support of the reference center in specialties. The monitoring of these patients is performed through home visits for the specific purpose of DOT. It is important to highlight that, in the context of the COVID-19 pandemic, there were some irregularities, especially when it comes to DOT, with a reduced frequency of visits and a shortage of professionals. As for routine consultations, some had to be rescheduled in order to minimize exposure to SARS-CoV-2.

It is possible to observe that this is a large municipality with an estimated population of 604,82 inhabitants, with the majority of the population (99.7%) residing in an urban area and having access to basic sanitation and drinking water. TB in the city under study has an incidence of 26.45 cases for every 100,000 inhabitants, 9.3% of patients with abandonment of treatment, and 63% of the proportion of new cases of pulmonary TB undergoing DOT. When it comes to coverage of primary health care, the municipality has a rate of 63.9%, and as for the coverage of the family health strategy, the index has only 21.5%, being an important predictor of MDR-TB [10].

### Participants

For the selection of participants, contact was made with the Tuberculosis Program of the Epidemiological Surveillance Center of the state of São Paulo for the presentation of the research project. After agreeing, we made contact with the coordination of the Program for the Control of Tuberculosis in Ribeirão Preto and the Infectious Diseases Sector of HCFMRP. Meetings were held with the managers of both organizations to identify key informants and plan data collection. Subsequently, a list of consensuses among the participating organizations was made available, containing the contacts of key professionals in the study.

It was adopted as an inclusion criterion, professionals who work or have worked directly in the monitoring of patients with MDR-TB. Thus, 14 health professionals with ties to the health system of Ribeirão Preto participated in the study (four doctors, three nurses, three nursing technicians, three nursing assistants and a social worker). Nursing technicians and nursing assistants are professionals with technical training who provide nursing services under the supervision of a nurse. Only one social worker was selected, because she is part of the city’s TB care network, providing direct follow-up to these patients. The duration of the activities of these professionals in the care of TB varied between 5 and 28 years, which shows important experience.

### Sampling and non-participation

Participants were recruited by purposive-convenience sampling of the TB care network of Ribeirão Preto [25]. In the selection of the sample, 18 health professionals were contacted, four of whom refused to participate, of these four, two did not answer the contact e-mails and the other two reported being overloaded in their work environments due to being on the front line fighting the pandemic. The minimum number of subjects defined for the study was 14, with the need to contemplate informants with homogeneous characteristics, conforming to a smaller sample of smaller dimensions, as evidenced by classic studies in the area [26].

### Interviews

Contacts with the health professionals were initiated, with a view to consenting to participate in the study and scheduling the interviews. From this, data collection was started by the main researcher who received previous training to approach the subjects, based on references in qualitative research [27].

The interviews guided through semi-structured scripts, as shown in Table 1, were carried out between June 2020 and August 2020, where professionals were able to discuss their experiences based on the main focus exposed by the researcher [28]. For Minayo [27], the interview privileges obtained information through individual speech, which reveals structural conditions and value systems, as well as transmits representations of a certain group through a spokesperson.

**Table 1:** Semi-structured script of interviews with health professionals

KEY QUESTIONS	
1. What is a normal day at your work like? What actions do you take? Can you describe a little of your activities in assisting patients with resistant TB? How much time in this role?	
2. When talking about MDR-TB, what are its causes?	
3. How has your experience been with caring/assisting patients with MDR-TB? What were the difficulties encountered?	
4. In your personal experiences, can the economic, social and educational levels of patients influence the illness? What have you experienced in your daily life?	
5. Can the difficulty of patients’ access to the health system contribute to the development of MDR-TB? Do you think that the organization of services can influence the treatment and development of MDR-TB?	
6. In your personal experience, have conditions, such as chronic diseases, alcohol consumption, smoking or use of other substances, influenced this outcome?	
7. Are health services prepared to deal with the problem of MDR-TB?	
8. Does the social protection network (family grant, social benefits, and basic food basket) impact the individual with MDR-TB? How does the presence or absence of these benefits influence MDR-TB cases?	
9. In your opinion, what can be done to prevent MDR-TB cases in the state of São Paulo?	
10. In your opinion, how would an assistance network for people with MDR-TB be provided?	
11. Considering the COVID-19 pandemic, do you think this will interfere with the morbidity and mortality picture from MDR-TB? Why?	
12. In your opinion, what advances have been achieved so far for the control of MDR-TB?	

The interviews lasted an average of 40 min and were conducted through an online tool and telephone calls using an audio recording application, following WHO’s recommendations for social distance as a result of the COVID-19 pandemic. During the interviews, the researcher made some records that were considered reflections for the study.

### Trustworthiness of the findings

In order to ensure the reliability of the results, systematic literature mapping was performed using the MEDLINE and Medical Literature Analysis and Retrieval System online databases via PubMed (US National Library of Medicine), Virtual Health Library (VHL) and Scopus, using the following descriptors: “Tuberculosis, Multidrug-Resistant,” “Health Personnel,” and “Health Services,” with the objective of identifying the scientific evidence that addresses the possible causes and/or social determinants of MDR-TB from the perspective of health professionals.

Thus, it was possible to verify that the social factors in the context of patients is the most prevalent determinant, inferring a direct relationship between MDR-TB and poverty. In addition, the issue of alcohol and drug abuse was frequently cited in which concerns were raised by professionals, reporting that drug abuse becomes a barrier to effective monitoring and adherence to treatment, in addition to interfering with the regularity of consultations, which may consequently limit the effectiveness of the follow-up and counseling process carried out by health professionals. In addition, the issue of stigma was raised, mainly by vulnerable groups, such as drug and/or alcohol users, migrants, people living on the streets and ethnic minorities, resulting in new barriers to access to health care.

### Data analysis

The interviews were transcribed in full. From that point on, the data were analyzed using the thematic content analysis technique [17]. The steps used to treat the data were the pre-analysis where the reading of testimonies was performed, which constituted the corpus of the analysis, followed by the exhaustive reading of the material. During the analysis, the record units (phrases) were selected, forming a section of the reports and subsequent organization. Next, the thematic grid was organized, from which the following thematic categories emerged: “the impacts of social determinants of health for the occurrence of MDR-TB,” “strategies for adherence and achieving equity,” and “challenges for adherence and equity in the context of the COVID-19 pandemic.” Table 2 contains the main issues addressed in the information allocated in the analytical categories, which are those that historically retain fundamental social relations for the knowledge of the object in general aspects and empirical categories are those built with operational exercise, managing to apprehend as determinations and as specificities that are expressed in empirical reality [27]. Table 2 shows the main synoptic categories of the key issues addressed in the categories.

**Table 2:** Synoptic table of the categories of key questions addressed in the interviews

Analytical categories	Empirical categories
The impacts of social determinants of health for the occurrence of MDR-TB	- High social vulnerability; - Homeless people; - Deprived of liberty; - Low educational level; - Inadequate food; - Legitimate drugs and illicit drugs
Strategies for adherence and achieving equity	- Social benefits as a way to improve nutrition; - Social benefits like “bargain” for treatment; - Basic basket as a facilitator for the treatment; - Financial assistance for transport; - Directly observed treatment.
Challenges for adherence and equity among patients in a context of the syndemic brought on by COVID-19	- Patients with MDR-TB may be more susceptible to COVID-19; - COVID-19 as an obstacle to the follow-up of MDR-TB treatment.

In order to guarantee the anonymity of the research participants, in the presentation of the statements, the letter “P” was used to refer to physicians, “N” for nurses, “NT” for nursing technicians, “NA” for nursing assistants and “SW” for social worker, followed by a numerical sequence from one to 14 in the order in which the changes were made.

## Results

### The impacts of social determinants of health for the occurrence of MDR-TB

Regarding the category, most professionals reported how DSS directly interfered in the treatment and follow-up of these patients with MDR-TB. Thus, the following sentences elucidate the issue of social classes, considering that those in lower positions tend to suffer more painfully from MDR-TB. In addition, some interviews were unable to distinguish the fact that some patients were drug users from being on the street, also making a parallel with the situation of social vulnerability in which they find themselves.

“Clearly, there are population groups that are more likely to get sick, and the main difference has to do with the socioeconomic condition, housing, education, and health. This is classic. Everyone knows this.” (P.2).

“Sometimes multi-resistant returns are people from a very low social level; unfortunately, they end up not adhering to the treatment.” (NT.8).

“Often they are patients who live on the street or are drug addicts, and they don’t have much concern about taking care of themselves. This ends up influencing the treatment.” (NT.9).

### Adherence strategies in the treatment of MDR-TB and achieving equity

In this category, it was sought to know which strategies were used by professionals to ensure greater adherence to treatment for patients with TB. Professionals reported that what keeps patients on the treatment is that they are receiving some kind of social benefit. Therefore, the success of treatment is directly related to the social protection system, because when he or she receives a benefit from social protection, he or she becomes more adherent to the treatment. With this, the health professionals reported that social benefits, such as transport assistance, food improvement, or food baskets, which were suspended by the municipal government during the COVID-19 pandemic, are strategies that helped patients in this process. Another strategy mentioned by health professionals is DOT; however, they also reported that there was a decrease in visits, resulting in guidance by health professionals to carry out the treatment in a self-administered way.

“He is very calm when he has some social assistance. He is more adherent because he does not want a problem at all. It is a negotiation like this.” (NA.12).

“The basic food basket helped us a lot, the so-called ‘bargain.’ If you do this, you come here to get the basket, then if you go to HCFMRP, collect the exams, pass an appointment, and take the medication every day for a certain time you have the right to a basic food basket. We used to tie this a lot.” (NA.11).

“When we had a basic basket, it was a bargaining chip. We said, ‘If you take it, if you do it, there will be a basket,’ that for them was the maximum, unfortunately.” (SW. 14).

The research participants stated that the coverage of social protection during the treatment of MDR-TB became an extremely important factor for the monitoring of these patients. Everyone believes that this contributes positively to better assistance and greater success with a positive outcome.

### Challenges for adherence and equity among patients in a context of the syndemic brought on by COVID-19

It was identified that most professionals we interviewed saw COVID-19 as an extremely important factor in interfering with the treatment of patients being followed up for MDR-TB. In general, the health professionals interviewed mentioned COVID-19 as an aggravating factor in the treatment of MDR-TB. Another factor noted as aggravating the adherence to treatment of patients with MDR-TB in the context of the pandemic was the irregularity of DOT, since this follow-up was done once a week in a self-administered way, which is not the ideal path (i.e., these patients under constant supervision), as this is a treatment that is difficult for the patient to adhere to.

“It is a conclusion, since the literature is indicating that the patient with [TB] is a patient who has a serious comorbidity because the shock organs are the lungs where the virus is located.” (P.4).

“The issue of [DOT], of being supervised for the time being, is once a week on behalf of COVID-19. The lack of professionals had a very big impact, because it was daily and now it is weekly.” (NA.11).

“Yes, the pandemic unfortunately hindered a lot, because DOT is self-administered sometimes. The person doesn’t care, they don’t want to take the medicine, and they won’t take it on their own. It’s very complicated.” (NT.10).

According to the empirical material and its relationship to the theoretical framework defined for the study, the three axes that conformed were not independent, keeping a relationship between them. We observed that, according to the reports, the DSS of MDR-TB was related to the socioeconomic structural determinant (income, unemployment, low education, and state of extreme poverty). These, in turn, are operating through intermediary determinants that are the material conditions of diagnosed patients’ lives (food insecurity, material living conditions, inadequate living conditions, unhealthy habits, and circumstances of the territory). It is possible to observe the determinants as an impact for the condition of multi-resistance (income, housing, material circumstances of the territory and neighborhood). According to the empirical material, the reports inform on the strategies used to improve treatment adherence, such as materialization of an investment in technologies, and care strategies, such as DOT, social protection and intersectoral actions, which are compensatory policies aimed at addressing inequalities in the territory to advance equity. With the COVID-19 pandemic, respondents reported that patients with MDR-TB were more susceptible to COVID-19 and consequently to a negative outcome due to already poor health conditions, considering COVID-19 as a stressor for monitoring the treatment of patients, so the pandemic situation negatively influenced the treatment and follow-up of patients with MDR-TB.

## Discussion

The study aimed to understand the perceptions of health professionals about MDR-TB and the strategies adopted to improve treatment adherence in individuals affected by COVID-19 in the state of São Paulo, Brazil. The findings revealed the issues that permeate a SDH and also the strategic mechanisms adopted to achieve adherence, with COVID-19 as an important stressor.

It is important to highlight that the professionals expressed great concern at the meeting between MDR-TB and COVID-19, since the situation of the pandemic has interfered in all the dynamics of patients diagnosed with MDR-TB. If the treatment of MDR-TB is interrupted, there are chances of dissemination of the disease. MDR-TB, which can make it a difficult to control situation, also weakens the capacity of the response to confront MDR-TB.

Regarding the approach of professionals to understand MDR-TB, it must be said that they are extremely important actors, because they have a broader view of the problems and difficulties that are encountered in carrying out the monitoring of patients with MDR-TB. However, many times they are powerless in the face of the need to solve problems for their patients.

However, this impotence often reported by professionals is due to a narrow view of the problem faced, because when these professionals report the problem according to their perceptions, some value judgments can be seen in their statements that can be attributed to prejudices. Many professionals report the fact that the patient has progressed to MDR-TB is often due to the social class in which he or she is inserted, stating that low-income population groups are more likely to fall ill. Knowing that poverty is one of the most important determinants of TB, it can also worsen the health condition of individuals [29]. Thus, TB and poverty maintain a dependent relationship, as poverty can be associated with precarious health conditions. Also, these two can produce poverty by reducing opportunities for work and subsistence [30].

Therefore, considering these aspects, it is necessary to raise awareness and humanize health professionals who deal daily with people in situations of social vulnerability, so discrimination and prejudice in services are not hindering access and care for people who need this more welcoming and integrated look [31]. Considering that this population needs to be empowered with regard to their rights, it is therefore important for health professionals to be facilitators of this process of learning, promotion and health education, strengthening bonds that make it possible to humanize the meeting. Therefore, it is necessary to understand without judging while respecting and establishing limits [32].

In addition, there is a lack of adherence to treatment, according to the reports, and respondents link non-adherence to the fact that these patients are homeless and/or drug users. Consequently, poverty has a direct influence in these cases, leading to abandonment. There is a relationship between these factors because the street environment can lead to the use of drugs, so consumption can provide a way of belonging to the street group, possibly composing survival strategies as the effects of the use of these substances produce sensations of pleasure, euphoria and power against the painful external reality, which leads to living in this context [33].

The interviews also show that health professionals somehow blame these individuals for not complying with the treatment, despite superficially considering the living conditions in which they are inserted. According to the literature [34], treatment adherence is a multidimensional phenomenon associated with factors, such as the conception of the disease, the treatment itself, and the relationship between the health system and professionals. In order to minimize the dropouts, adherence strategies in the treatment of MDR-TB were presented by most of the interviewees, an example of which are the benefits that were offered to this population. The social protection that covers patients with TB can provide a means for them to reduce treatment defaults, especially for the poorest [29]. A meta-analysis carried out with data from nine randomized clinical trials involving 1687 participants showed that social protection strategies improved access to health care and, consequently, adherence to TB treatment [29]. For these interviewed professionals, this question was seen as something positive to maintain regular follow-up, as these actions enable access, the bond that they often use as a subsidy for the adequate treatment.

However, a weakness brought to the study is that the regular monitoring of patients with MDR-TB has been hampered by the COVID-19 pandemic, aggravating a scenario that was already very complex because of inequality and poverty. Instead, the health professionals redesigned DOT, and to advance in terms TB control, the professionals had to reduce home visits for DOT due to the lack of professionals and subsidies, which was an emblematic contradiction under the care perspective. The interpersonal relationship that occurs between patients and health professionals in performing DOT is of paramount importance for the motivation and monitoring of treatment, assessment of the risk of abandonment, and also for the management of side effects; however, interruptions in this process can affect the continuity of patients.

Santos and colleagues [35] conclude in their study that the influence of socioeconomic factors on adherence to monitoring and treatment of MDR-TB is evident even when the treatment is free, and the absence of social benefits can hinder treatment continuity. However, an offer of such assistance requires that selected health services be more integrated with other social assistance equipment and exemplifies the relationship of organizational and economic accessibility.

In view of this, a technical dimension of the services must be added, which focuses on their adequacy to the clients’ needs and the quality of services; a dimension focused on interpersonal relationships that observe the psychological and social interaction between patients and health professionals and finally an organizational dimension of the process regarding accessibility to services and the extent of coverage of the services offered, encouraging therapeutic adherence and reducing treatment dropout, in addition to helping patients to reframe their life projects [36].

Thus, new conformations of the health system and care practices are being considered in the context of the pandemic, with emphasis on the implementation of Web-DOT, which enables remote monitoring of patients with TB and guarantees this adherence [37], as well as mobilizing family volunteers to supervise these patients under treatment.

The study also showed a great concern of professionals with the worsening of the patient’s health condition, as they already have MDR-TB and also contracted COVID-19, due to the condition of the lung already being weakened by MDR-TB. There is evidence that the meeting of these two diseases or even the simultaneous occurrence of phenomena will be a “catastrophe” [5], which some epidemiologists classify as a true syndemic, given the contexts of social inequality and deprivation [38]. Furthermore, no patient was reported in this condition, but there was great effort on the part of professionals to prevent the situation.

In the literature, it is observed that therapeutic discontinuities also result from the form of organization of health services, which sometimes has an incompatible functioning agenda. For example, from a patient who is working in formality and/or informality [39], the requirement for the patient to visit the health center, which is sometimes very far from home, without any financial support from the health system that also requires an approach with this actor.

The recognition of TB control and elimination is linked to the debate on health promotion, which is based on healthy public policy proposals and intersectorality. Intersectorial action involves the creation of communicative spaces, the ability to negotiate and also the overcoming of conflicts so that one can finally reach actions that mainly involve the accumulation of molds, constructions of formulations and discoveries of possibilities for action [40]. The intersectorial approach leads us, in turn, to the understanding of social support, which runs through the following categories: spouse, family, friends, neighbors, colleagues, specialized services, self-help groups and professionals of health and wellbeing [41].

The elimination of TB runs through the question of understanding the determinants of MDR-TB in the light of different perspectives, including that of professionals who are on the front line of care. The issue of MDR-TB was already a challenge in the country due to the low percentage of adherence, and it could intensify with the pandemic. However, the study showed a commitment on the part of professionals to face the situation and to achieve equity in care.

In this way, the limitations of the study refer to the study’s online approach, which may have prevented researchers from being able to capture other forms of communication (e.g., non-verbal). It would be of great importance for future studies to include patients diagnosed with MDR-TB in order to carry out an investigation on both sides of the therapeutic relationship. However, the study brought relevant points for qualification and development of services, being one of the first, which has been working with MDR-TB in the context of the pandemic.

The study has used a more ontological and interpretivism perspective, searching for reasons, meanings, and a certain inter-subjectivity between providers and patients. There was not concern with the generalization of the findings into other contexts or scenarios. The meanings and beliefs brought in the study evidenced DOT was very limited in terms of motivating and reframing the patients into their life projects and self-esteem. Even covered by DOT, the providers revealed feeling fear and insecurity manifested by the patients, which was a big contradiction in the findings. DOT should have ensured that they were self-confident. The incorporation of new knowledge is really necessary to implement a more qualified DOT, which must ensure not only the continuity of the treatment in a biomedical perspective but also something more valuable for patients that is their lives. They should be motivated to continue their treatment even in a context dramatically affected by COVID-19, without prejudices or stigma.

## Conclusion

The study advances knowledge by highlighting the challenges faced by health professionals working in the health system for adherence to treatment and monitoring of patients diagnosed with MDR-TB in the social context aggravated by the COVID-19 pandemic. The strategies defined by these health professionals, to some extent, have guaranteed the achievement of equity, avoiding the catastrophic encounter between MDR-TB and COVID-19.

## Data Availability

The data contains potential identification of each study participant, such as full name, date of birth, age and locale of residence. In addition, data were collected from a small group of participants in a priority city in Brazil. All participants were informed about the objectives of the study and how their confidentiality would be protected, as well as their right to withdraw from the study at any time. The participants included in this study gave their consent to use only anonymized quotes in this research. All relevant data are within the paper. The raw individual participant’s interview transcripts are not publicly available due to confidentiality and privacy concerns. The first author has registered their details, as well as contact data in case of interest in collaborative work or further information.

## Abbreviations

COREQ: Consolidated Criteria for Reporting Qualitative Research
DOT: Directly Observed Treatment
HCFMRP: Clinics Hospital of the Ribeirão Preto Medical School at University of São Paulo
MDR: Multidrug-Resistant
MDR-TB: Multidrug-Resistant Tuberculosis
SDH: Social Determinants of Health
TB: Tuberculosis
WHO: World Health Organization
